# Current use of fluid biomarkers as outcome measures in Multiple Sclerosis (MS): a review of ongoing pharmacological clinical trials

**DOI:** 10.1007/s10072-023-07228-3

**Published:** 2023-12-20

**Authors:** Edoardo Dalmato Schilke, Giulia Remoli, Eugenio Funelli, Michela Galimberti, Maria Letizia Fusco, Diletta Cereda, Claudia Balducci, Maura Frigo, Guido Cavaletti

**Affiliations:** 1 Neurology Department, Fondazione IRCCS San Gerardi dei Tintori, Monza, Italy; 2 School of Medicine and Surgery and Milan Centre for Neuroscience (NeuroMI), University of Milano-Bicocca, Milan, Italy

**Keywords:** Multiple sclerosis, Fluid biomarkers, Clinical trials, Neurofilament light chains

## Abstract

The present study aims to describe the state of the art of fluid biomarkers use in ongoing multiple sclerosis (MS) clinical trials.

A review of 608 ongoing protocols in the clinicaltrials.gov and EudraCT databases was performed. The trials enrolled patients with a diagnosis of relapsing remitting MS, secondary progressive MS, and/or primary progressive MS according to Revised McDonald criteria or relapsing MS according to Lublin et al. (2014). The presence of fluid biomarkers among the primary and/or secondary study outcomes was assessed.

Overall, 5% of ongoing interventional studies on MS adopted fluid biomarkers. They were mostly used as secondary outcomes in phase 3–4 clinical trials to support the potential disease-modifying properties of the intervention. Most studies evaluated neurofilament light chains (NfLs). A small number considered other novel fluid biomarkers of neuroinflammation and neurodegeneration such as glial fibrillary acid protein (GFAP).

Considering the numerous ongoing clinical trials in MS, still a small number adopted fluid biomarkers as outcome measures, thus testifying the distance from clinical practice. In most protocols, fluid biomarkers were used to evaluate the effectiveness of approved second-line therapies, but also, new drugs (particularly Bruton kinase inhibitors). NfLs were also adopted to monitor disease progression after natalizumab suspension in stable patients, cladribine efficacy after anti-CD20 discontinuation, and the efficacy of autologous hematopoietic stem cell transplant (AHSCT) compared to medical treatment. Nevertheless, further validation studies are needed for all considered fluid biomarkers to access clinical practice, and cost-effectiveness in the “real word” remains to be clarified.

## Introduction

Multiple sclerosis (MS) is a chronic inflammatory demyelinating disease involving the central nervous system (CNS) resulting from the interaction of genetic and environmental factors that are only partially understood. Clinical symptoms vary based on the anatomical location of lesions and often correlate with the invasion of inflammatory cells across the blood-brain barrier (BBB) with consequent demyelination and edema [1]

Although the course is highly variable, the development of irreversible disability represents the natural course of the disease. MS remains a notable cause of neurological disability in young adults [2, 3]. Together with neurological symptoms and signs, the spatial and temporal distribution of inflammatory lesions revealed by magnetic resonance imaging (MRI) is the primary diagnostic feature [3]. However, MS specificity remains an issue, with many patients needing to satisfy established diagnostic criteria delaying proper disease management and treatment [4].

Thus, in recent years, there has been considerable effort in finding more accurate biomarkers to rapidly identify disease processes and different MS trajectories and differentiate them from other neurological conditions. Given the inter-relation with the blood-brain barrier (BBB) and brain parenchyma, cerebrospinal fluid (CSF) was the primary object of new biomarkers investigation [4]. The main benefit of using CSF over blood to measure biomarkers was that it more accurately reflects the inflammatory profile within CNS [5].

Accordingly, the immunoglobulin G (IgG) index and oligoclonal biomarker detection have been systematically validated as CSF biomarkers for multiple sclerosis.

As increasingly sensitive technological platforms are being developed, the feasibility of identifying soluble biomarkers in blood has improved, as supported by the role of the neurofilament light chain (NfL) in serum and plasma for evaluating response to therapy and disease activity [4].

CSF biomarkers benefit from being more sensitive than clinical or radiological assessments, especially in low-grade MS activity [5].

Indeed, among those patients whose disease was inactive according to clinical scales and/or MRI, CSF neurofilament light chain (cNfL) and immunoglobulin (Ig)G index were significantly elevated. Furthermore, some studies demonstrated that intrathecal IgG synthesis was a hallmark of MS and IgM synthesis: increased IgM index or detection of OCMB (Oligoclonal IgM bands) was an unfavorable diagnostic marker [6]. Indeed, OCMBs are associated with increased MS activity (i.e., increased retinal axonal loss, decreased retinal nerve fiber layer, and more aggressive disease progression during the early stages of relapsing-remitting multiple sclerosis, RRMS). The presence of oligoclonal bands in CSF is predictive of conversion from CIS to MS [5]. Over the past three decades, many assays have been developed to detect neurofilament light chain (NfL) levels [7]; several features make NfL, cytoskeletal proteins released from damaged axons into the CSF and the blood, a promising biomarker of neurodegeneration.

NfL can be objectively measured and quantified: over the last few years, a single molecular array (Simoa) has made measuring NfL concentration levels more reliable and clinically relevant [4].

Several studies showed that NfL levels increase during MS relapses and correlate with MRI lesion volume [8, 9], disease activity, disability, and disease progression [10]. In addition, a growing body of evidence reported that NfL in cerebrospinal fluid (CSF) and serum could be used as reliable indicators of treatment response.

Other novel biomarkers seem promising in MS. A recent meta-analysis of studies confronting CSF glial fibrillary acid protein (GFAP) levels in MS patients compared to healthy controls has shown significantly higher levels in MS patients. Progressive MS patients tendentially had higher CSF GFAP concentration than RRMS [11]. Additionally, higher levels of GFAP have been associated with greater disabilities and shorter relapse intervals [4].

There is growing research interest in inflammation biomarkers. According to recent findings, immune signatures assembled with the markers mentioned above could further differentiate underlying disease pathology and disease activity. Chitinase-3-like-1 precursor (Chi3L1) is a glycoprotein secreted by various cell types, including activated astrocytes and microglia [12]. It has been found to be increased in CSF of patients with different inflammatory diseases of the central nervous system. In MS, higher CSF levels have been associated with a more rapid conversion to RRMS in CIS patients; lower CSF levels have been found in progressive MS compared with RRMS [13]. The same data was not confirmed on plasma where Chi3L1 levels resulted higher in progressive patients. Otherwise, according to what was found in CSF, plasma levels resulted associated with more radiological relapses [14], and serum levels were associated with a more rapid conversion from CIS to RRMS [13]. In another study, serum Chi3L1 levels were found to increase in groups of patients unresponsive to b-interferon therapy [15]. C-X-C Motif Chemokine 13 (CXC13) is a chemokine protein-ligand [16]. High CSF CXCL13 concentration was confirmed in MS by several studies and associated with an increased risk of clinically definite MS and a more severe disease course in RRMS, SPMS, and PPMS patients [17–20]. Not surprisingly, CSF CXCL13 levels seem to be a robust and sensitive indicator of intrathecal B-cell response, even in the presence of an intact blood-brain barrier [21].

Serum pro-inflammatory cytokine IL-6 has been found to be correlated with the age of onset of MS, and higher levels were detected in MS patients compared to controls [22]. Serum levels of the anti-inflammatory cytokine IL-10 were inversely correlated with the risk of relapse in pediatric MS [23].

Despite the growing research interest in plasma and CSF biomarkers of MS, their use in clinical trials and prospective studies still seems limited. The study aimed to review and summarize the state of the art of biomarkers implementation in ongoing MS study protocols registered in clinicaltrials.gov and EudraCT databases. The review was focused on neuroinflammation- and neurodegeneration-related biomarkers according to the body of literature available in our query.

This analysis may inform how MS biomarkers are adopted in drug development, shedding light on potential methodological, clinical, and ethical issues. Furthermore, it can provide reflection points on how biomarkers are translated from a pure research context to a clinical one.

## Materials and methods

### Data source and search strategy

Two databases were used as reference sources for the present review: (i) the *ClinicalTrials.gov* for studies registered in the USA and (ii) the *EudraCT* (European Union Drug Regulating Authorities Clinical Trials Database) for all interventional studies registered in the European Union.

*Clinicaltrials.gov* is an online free database provided by the U.S. National Library of Medicine, gathering information from privately and publicly funded clinical studies conducted worldwide on a wide range of diseases and conditions. *EudraCT* is an online free database for all interventional clinical trials on medicinal products submitted to the National Competent Authorities (NCAs) of the European Union.

The two databases were lastly investigated on *May 2023* by using the following terms and fields in the advanced search function on *Clinicaltrials.gov* : “multiple sclerosis OR MS” [CONDITION OR DISEASE] AND “interventional studies (clinical trials)” [STUDY TYPE] AND (“not yet recruiting” OR “recruiting” OR “enrolling by invitation “OR “active, not recruiting”) [STATUS: RECRUITMENT] AND (“phase 1” OR “phase 2” OR “phase 3” OR “phase 4”) [PHASE]. On *EudraCT*, selecting specific search functions is impossible.

There was no restriction on age, sex, date, and location.

Two authors (E.D.S + G.R; M.G + E.F) independently screened the identified records to remove duplicates and verify the fulfilment of the following predefined inclusion criteria:

(i) Targeting subjects with clinical disturbances associated within the MS continuum (e.g., CIS; RRMS; PPMS)

(ii) Testing the safety, efficacy, or tolerability of pharmacological interventions.

**Fig. 1:**
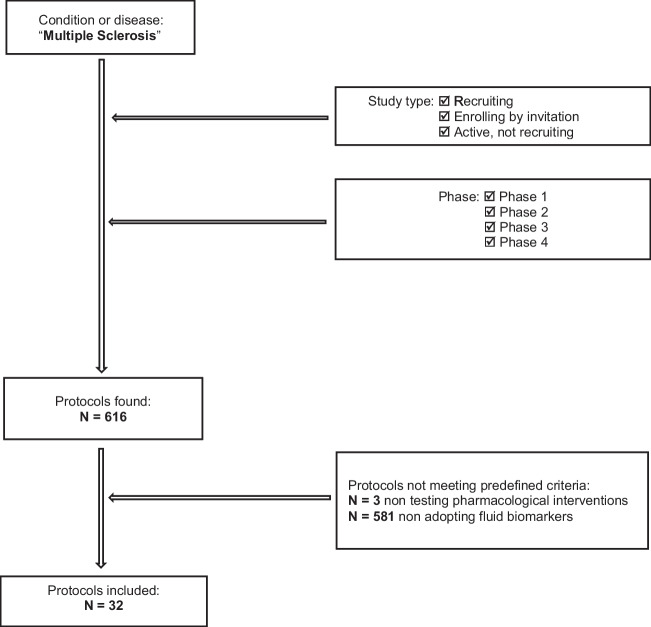
Flow chart of protocols selection

The flow chart (Fig. 1) shows the selection of the protocols of interest for the present review.

### Data extraction and analysis

Data were extracted from trials included in this review using extraction tables. Four reviewers (G.R + E.D.S and E.F + M.G) independently extracted the following data from the selected protocols:

i. NCT number
ii. Phase
iii. Study design
iv. Expected end date
v. Location
vi. Planned number of participants
vii. Pharmacological intervention
viii. Age
ix. Diagnosis
x. Expanded disability status score (EDSS)
xi. Adopted diagnostic criteria
xii. Main criteria of exclusion/inclusion
xiii. Fluid biomarkers adopted as either primary or secondary outcome measure (s).

Disagreements in the selection process and data extraction were solved by consensus or involving two additional reviewers.

## Results

In Table 1 are listed NCT number, phase, study design, expected end date, and location of selected protocols; in Table 2 are listed NCT number, planned number of participants, pharmacological intervention, age, diagnosis, EDSS, adopted diagnostic criteria, main criteria of inclusion/exclusion, and fluid biomarkers adopted as either primary or secondary outcome measures of selected protocols. Lastly, Table 3 describes how fluid biomarkers were adopted according to MS phenotype.

**Table 1:** Listed NCT number, phase, study design, expected end date, and location of selected protocols

NCT	Phase	Study design	Expected end date	Location
NCT05349474	1	Double-blinded RCT	May-26	USA
NCT05417269	2	Double-blinded RCT	Dec-25	Moldova
NCT03979456	3	Single-blinded RCT	Jun-25	Sweden
NCT05630547	2	Quadruple-blinded RCT	Aug-25	Multi-country
NCT04121403	3	Single-blinded RCT	Dec-24	Norway
NCT04544436	3	Double-blinded RCT	Aug-28	USA
NCT05147220	3	Double-blinded RCT	Oct-30	USA
NCT0454899	3	Double-blinded RCT	Jul-29	Canada
NCT04926818	3	Quadruple-blinded RCT	Jun-29	USA
NCT04695080	3	Quadruple-blinded RCT	Dec-26	UK
NCT04410991	3	Triple-blinded RCT	Aug-23	USA
NCT04510220	3	Open-label	Dec-21	USA
NCT04586023	3	Double-blinded RCT	Nov-25	USA
NCT03963375	4	Open-label	May-25	USA
NCT03193866	4	Observational	Dec-22	Sweden
NCT05296161	4	Open-label	Mar-26	Netherlands
NCT05232825	3	Open-label	Apr-25	USA
NCT04458051	3	Triple-blinded RCT	Aug-24	USA
NCT04411641	3	Double-blinded RCT	Aug-24	Multi-country
NCT03650114	3	Open-label	Mar-30	USA
NCT04047628	3	Single-blinded RCT	Oct-29	USA
NCT04688788	3	Single-blinded RCT	Apr-28	Denmark
NCT04338061	3	Quadruple-blinded RCT	Jun-26	USA
NCT02688985	3	Open-label	Jul-23	USA
NCT04550455	4	Open-label	Dec-25	USA
NCT04640818	4	Observational	Oct-22	Switzerland
NCT05090371	4	Open-label	Nov-25	USA
NCT04048577	4	Open-label	Dec-21	USA
NCT04239820	4	Observational	Jun-24	Finland
NCT04877457	4	Quadruple-blinded RCT	Jul-28	USA
NCT03540485	2	Double-blinded RCT	Dec-24	Spain

**Table 2:** Listed NCT number, planned number of participants, pharmacological intervention, age, diagnosis, EDSS, adopted diagnostic criteria, main criteria of inclusion/exclusion, and fluid biomarkers adopted as either primary or secondary outcome measures of selected protocols

NCT	Patients number	Molecule	Age	Diagnosis	EDSS	Diagnostic criteria	Main criteria	Primary outcome	Secondary outcome
NCT05349474	44	Metformin	30–65	PPMS SPMS	0–9.5	McDonald 2017	Stable in the last 6 months	-	sNfL levels at baseline and after 12 months
NCT05417269	150	IMCY-0141 vs dimethylfumarate vs placebo	18–45	RRMS	0–5	McDonald 2017	Treatment-naive disease duration ≤ 3 years	-	sNfL at baseline and after weeks 2, 4, 6, 8, 10, 12, 24, and 36.
NCT03979456	200	Rituximab 500mg /6 months vs. Rituximab 500mg /12 months	20–52	RRMS CIS	0–5.5	McDonald 2017	Completed the RIFUND-MS trial	-	Mean change of sNfL concentration between the two dosing arms
NCT05630547	168	SAR443820 vs placebo	18–60	MS	2–6	McDonald 2017	Untreated/stable patients	sNfL levels at 48 and 96 weeks	
NCT04121403	264	Rituximab vs Cladribine	18–65	RRMS	0–9.5	McDonald 2017	Disease activity in the last 12 months	-	sGFAP at week-1, 51, and 96 weeks sNfL at week-1, 51, and 96 weeks
NCT04544436	865	Ocrelizumab 1200/1800 mg /24 weeks vs Ocrelizumab 600 mg /24 weeks	18–55	RRMS, aSPMS	0–5.5	McDonald 2017	≥2 clinical relapses within the last 2 years or ≥1 clinical relapse in the last year No relapse 30 days prior to screening	-	bNfL from baseline up to 4.3 years bIL-6 from baseline up to 4.3 years
NCT05147220	800	Remibrutinib vs Teriflunomide	18–55	RRMS	0–5.5	McDonald 2017	≥1 relapse within the previous year or ≥2 relapses within the previous 2 years Neurologically stable within 1 month	-	sNfL levels from baseline and up to years
NCT0454899	699	Ocrelizumab 1200/1800 mg /24 weeks vs Ocrelizumab 600 mg /24 weeks	18–55	PPMS	0–5.5	McDonald 2017	Neurologically stable within 1 month	-	bNfL from baseline up to 4.3 years bIL-6 from baseline up to 4.3 years
NCT04926818	180	Ofatumumab vs Fingolimod vs Siponimod	10–17	RRMS	0–5.5	McDonald 2017	≥1 relapse within the previous year or ≥2 relapses within the previous 2 years	-	sNfL at day 1 and after 3, 6, 12, 18, and 24 months
NCT04695080	200	Cladibrine vs placebo	> 18	AMS	6.5–8.5	McDonald 2017	Deterioration of upper limb function in the 2 years previous baseline No relapse within 6 months	–	sNFL at baseline, 6 and 24 months Association between sNFL and memory B count and upper limb function
NCT04410991	900	Tolebrutinib vs Teriflunomide	18–55	RRMS	0–5.5	McDonald 2017	≥1 relapse within the previous year or ≥2 relapses within the previous 2 years	-	pNfL from baseline and after 36 months sChi3L1 from baseline and after 36 months
NCT04510220	10	Ofatumumab	18–60	RRMS	0–5.5	McDonald 2017	≥1 relapse within the previous year or ≥2 relapses within the previous 2 years	-	sGFAP at 5, 28 90, 273 days and correlation with microglial activity sNFL at 5, 28 90, 273 days and correlation with microglial activity
NCT04586023	736	Fenebrutinib vs Teriflunomide	18–55	RRMS	0–5.5	McDonald 2017	-	-	sNfL from baseline and after 48 weeks
NCT03963375	50	Cladribine	18–65	RRMS	0–5.5	McDonald 2017	At least 1 relapse or 1 GD or 1 new or enlarged T2 lesion in the last 12 months	-	cNfL at 5 weeks, 10 weeks, 1 year, or 2 years
NCT03193866	3526	Rituximab vs other DMTs	> 18	CIS RRMS	0–9.5	McDonald 2017	Treatment naïve or second DMT of a different class	-	sNfL at baseline, 3, and 9 years
NCT05296161	296	B-cell tailored Ocrelizumab vs standard Ocrelizumab	18–60	RRMS	0–6.5	McDonald 2017	Treatment with ocrelizumab for a minimum of 48 weeks	-	sNfL at baseline and 96 weeks
NCT05232825	234	Ocrelizumab SC vs Ocrelizumab IV	18–65	RRMS PPMS	0–6.5	McDonald 2017	Neurological stability for ≥30 days	-	sNfL at day 1, weeks 12, 24, 48
NCT04458051	990	Tolebrutinib vs placebo	18–55	PPMS	2–6.5	McDonald 2017	Disease duration <15 years if EDSS >5.0 or <10 years if EDSS≤5.0.CSF OB + and/or elevated IgG index	-	pNfL from baseline up to 48 months pChi3L1 from baseline up to 48 months
NCT04411641	1131	Tolebrutinib vs placebo	18–60	RRMS SPMS	3–6.5	McDonald 2017	Documented disability progression within 12 months No clinical relapses for at least 24 months	-	pNfL from baseline up to 48 months pChi3L1 from baseline up to 48 months
NCT03650114	2060	Ofatumumab	18–100	RRMS	0–9.5	McDonald 2017	Completed a selected Novartis MS study which dosed ofatumumab every 4 weeks	-	sNfL from baseline up to 5 years
NCT04047628	156	AHSCT vs. BMT	18–55	RRMS SPMS	0–6	McDonald 2017	≥ 2 episodes of disease activity in the 36 months prior ≥ 1 episode of disease activity must occur following ≥ 1 month of treatment with an oral DMT	-	sNfL from baseline up to 72 months
NCT04688788	594	Rituximab vs Ocrelizumab	18–65	MS	0–6.5	McDonald 2017	Treatment naive RRMS or previously treated RRMS or PMS patients with clinical/radiological activity in the last year for PMS also s/cNfL elevation considered	-	sNfL from baseline up to month 24
NCT04338061	898	Evobrutinib vs Teriflunomide	18–55	RMS	0–5.5	McDonald 2017	≥1 documented relapses within the 2 years before screening	-	sNfL at baseline and after 12 weeks
NCT02688985	132	Ocrelizumab	18–55	RMS	0–5.5	McDonald 2010	Documented relapse in the past year	cNfL at baseline and after 12, 24, 52, 144, or 240 weeks	-
				PPMS	3–6.5		Elevated IgG Index or OCBs	-	cNfL in CSF baseline and after 12, 24, 52, 144, or 240 weeks
NCT04550455	30	Cladribine	21–65	SPMS	2–7	Lublin 2014	Neurologically stable for > 30 days prior	s/c NfL values from pre-treatment to week 48	cGFAP from baseline up to 96 weeks cNfL and sNfL antibody baseline and 96 weeks
NCT04640818	45	Cladribine after antiCD20 vs antiCD20	> 18	RMS	0–7	Lublin 2014	Treatment with antiCD20 for ≥18 months	-	Changes in sNfL from baseline to 12 months
NCT05090371	150	Ofatumumab after DMT vs. DMT	18–50	RRMS	0–5.5	McDonald 2017	On a DMT for at least 6 months No relapse reported within 6 months	-	% of participants with baseline NfL≥10pg/ml and NfL<10pg/ml achieving NEDA-3
NCT04048577	10	Interruption of Natalizumab vs maintenance of Natalizumab	21–65	RRMS	0–9.5	McDonald 2017	Clinically stable with pre-planned annual dose interruption of 2 consecutive doses	b/c NfL at interruption and after 6 months	sVCAM sMAdCAM at interruption and after 6 months
NCT04239820	15	Cladribine	45–55	RRMS	0–9.5	McDonald 2017	N	-	sNfL at baseline and 18 months sGFAP at baseline and 18 months
NCT04877457	100	Ocrelizumab	18–40	RIS	0	McDonald 2017	Diagnosis established within last 5 years	-	sNfL baseline and after 24, 48, 72, 104, 130, 156, 182, 208 weeks
NCT03540485	50	Melatonin + Ocrelizumab vs Ocrelizumab alone	18–65	PPMS	2–7	McDonald 2010	No immunomodulatory treatment in the prior 9 months No melatonin during the month prior to participation	-	sCXCL13 at baseline and after 48 weeks sNfL at baseline and after 48 weeks

**Table 3:** Describes how fluid biomarkers were adopted in clinical trials according to MS phenotype*. n*, number of clinical trials that adopted a candidate fluid biomarker as outcome measure for a definite MS phenotype. *RIS* radiologically isolated syndrome, *CIS*, clinically isolated syndrome; *RRMS* relapsing remitting MS, *SPMS* secondary progressive MS, *PPMS* primary progressive MS, *MS* multiple sclerosis patients, *AMS* active MS, *RMS* relapsing MS

Biological sample	Biomarker	MS phenotype							
		RIS	CIS	RRMS	SPMS	PPMS	MS	AMS	RMS
Blood	NfL	*n*= 1	*n*= 2	*n*= 15	*n*= 6	*n*= 4	*n*= 2	*n*= 1	*n*= 2
CSF	NfL	*n*= 0	*n*= 0	*n*= 2	*n*= 1	*n*= 1	*n*= 0	*n*= 0	*n*= 1
Blood	GFAP	*n*= 0	*n*= 0	*n*= 3	*n*= 0	*n*= 0	*n*= 0	*n*= 0	*n*= 0
CSF	GFAP	*n*= 0	*n*= 0	*n*= 0	*n*= 1	*n*= 0	*n*= 0	*n*= 0	*n*= 0
Blood	IL-6	*n*= 0	*n*= 0	*n*= 1	*n*= 1	*n*= 1	*n*= 0	*n*= 0	*n*= 0
Blood	Chi3L1	*n*= 0	*n*= 0	*n*= 2	*n*= 1	*n*= 1	*n*= 0	*n=* 0	*n*= 0
Blood	VCAM	*n*= 0	*n*= 0	*n*= 1	*n*= 0	*n*= 0	*n=* 0	*n=* 0	*n*= 0
Blood	MadCAM	*n*= 0	*n*= 0	*n*= 1	*n*= 0	*n*= 0	*n*= 0	*n*= 0	*n*= 0
Blood	CXCL13	*n*= 0	*n*= 0	*n*= 0	*n*= 0	*n=* 1	*n*= 0	*n*= 0	*n*= 0

### Search results

A total of 608 protocols of phase 1, 2, 3, and 4 interventional studies, retrieved by the structured search on clinicaltrials.gov and the EUDRA database, were screened. Overall, only in 5% (*n* = 32) of the protocols, fluid biomarkers were adopted as outcome measures.

Overall, 32 protocols were phase 3 (*n* = 18) and phase 4 (*n*= 9) trials. In 3 cases, fluid biomarkers were secondary outcome measures in phase 2 clinical trials; they were also adopted in a phase 1 clinical trial for an early assessment of metformin remyelinating properties in PPMS and SPMS patients.

### Demographics

In almost all protocols, the 2017 Revised McDonald Criteria were adopted for patients’ selection [24]. Two studies considered the 2010 Revised McDonald Criteria [25], whereas 2 protocols adopted the 2014 Lublin criteria to identify relapsing MS patients, independently from a relapsing-remitting or secondary progressive disease progression [26].

Most studies were conducted on adult RRMS patients; the age of patients recruited ranged from 18 to 100 years, but most protocols included patients from 18 to 65 years. One study was addressed to pediatric patients (from 10 to 17 years). RRMS patients included had mostly a baseline EDSS score from 0 to 5.5 or 6.5, although some trials included patients independently from baseline disability status. In 9 protocols, fluid biomarkers were adopted as outcome measures in SPMS and/or PPMS patients. The age of progressive patients included ranged from 18 to 65 years, and the EDSS score for inclusion was variable in the different studies. Finally, patients with other diagnoses were included in 7 protocols, namely, RMS (*n* = 3), MS (*n* = 2), active MS (*n* = 1), CIS (*n* = 1), and RIS (*n* = 1) patients.

### Interventions

Fluid biomarkers were prevalently considered in studies about the effectiveness of approved second-line therapies (ocrelizumab, rituximab, ofatumumab, cladribine, siponimod, and natalizumab) compared to other disease-modifying therapies or placebo. On the other hand, almost all clinical trials evaluating new drugs, particularly Bruton tyrosine kinase (BTK) inhibitors (remibrutinib, tolebrutinib, fenebrutinib, SAR443820), considered fluid biomarkers. In one trial of stable MS patients, NfLs were used to monitor disease progression after disease-modifying therapies suspension, particularly natalizumab; NfLs have also been considered to monitor cladribine disease modifying efficiency after anti-CD20 drugs discontinuation and to evaluate the efficacy of autologous hematopoietic stem cell transplantation (aHSCT) compared to best medical treatment. As mentioned above, 1 protocol evaluated metformin remyelinating properties in PPMS and SPMS patients.

### Fluid biomarkers

Four protocols indicated fluid biomarkers as primary outcomes and 32 as secondary outcomes, whereas in 1 case as both primary and secondary outcome measures.

The use of fluid biomarkers mostly relied on serum NfLs (sNfL): 18 protocols adopted sNfL as secondary outcomes and 2 protocols as primary outcome measures. NfLs were also evaluated in plasma (pNfL) in 3 protocols as secondary outcomes. Four protocols, which study design contemplated multiple successive lumbar punctures, evaluated NfL in CSF (cNfL); in one case, cNfL were considered as primary outcome measure. In 5 protocols, NfL were reported as generically tested in blood (bNfL).

Given a baseline determination at the start of the protocol, the mean change of sNfL concentration in subsequent time points was compared in the two dosing arms. However, 2 protocols considered the absolute difference of NfL concentration in subsequent time points between the two arms.

Some novel fluid biomarkers were also adopted. Serum GFAP (sGFAP) was a secondary outcome measure in 3 protocols; 1 study also considered GFAP in CSF (cGFAP). In all cases, GFAP was evaluated in combination with NfL. In one protocol, sGFAP and sNfL concentrations in subsequent time points were correlated with microglial activity, measured as standardized uptake value ratio (SUVR) of [F-18] PBR06 in brain PET scans.

Other fluid biomarkers adopted were blood IL-6 (*n* = 2), serum Chi3L1 (*n* = 2), serum VCAM (*n* = 1), serum MadCAM (*n* = 1), and serum CXCL13 (*n* = 1). In all cases, they were adopted as secondary outcome measures and in combination with NfL.

## Discussion

MS is a disease characterized by high clinical, radiological, and pathological features and therapeutic response heterogeneity. Therefore, there is an urgent need for reliable biomarkers to capture the varied aspects of disease heterogeneity. At now, this aspect is not reflected in clinical trials: only a few protocols included fluid biomarkers as outcome measures, and none have adopted them for participant eligibility criteria.

Most of the selected protocols adopted NfL as a surrogate endpoint of neuroaxonal damage, the culprit of disability development in MS. Table 3 indicates that NfL are extensively used in protocols as a biomarker across all MS phenotypes. While NfL indicate acute axonal damage determined by inflammation, neurofilament heavy chains (NfH) more effectively capture chronic axonal damage, strongly correlating with disability progression [27]. However, only NfL have been incorporated into clinical trials to date.

Numerous studies endorse using NfL to assess the effect of immunomodulatory treatments. In phase 3 β-interferon (IFN-β) clinical trials and its extension studies, a significant reduction of CSF NfL levels was observed in patients treated with IFN-β compared to those treated with placebo; an increase of NfL levels was also evidenced in patients with a suboptimal treatment response [28]. In a study of 32 treatment-naïve RRMS patients initiating either glatiramer acetate (GA) or IFN-β first decreasing and then afterwards consistently low NfL levels were documented in therapy-responsive patients. Otherwise, NfL levels stayed elevated and aligned with MRI relapse activity. [29]. Dimethyl-fumarate (DMF) effect on CSF, serum, and plasma NfL was assessed in 104 treatment-naïve RRMS patients receiving either treatment or placebo; after 1 year of treatment, DMF reduced CSF, plasma, and serum NfL levels were reduced to levels comparable to that measured in healthy controls [30]. Treatment with natalizumab for 60 weeks was also associated with a decrease in NfL level in CSF in a single-arm prospective cohort study. In this cohort, changes in NfL levels correlated with clinical improvement [31]. One longitudinal study evaluating 243 RRMS patients showed that fingolimod treatment decreased plasma NfL levels 12 and 24 months after baseline [32]. Compared to placebo, a decrease in blood NfL concentration was observed in ocrelizumab and fingolimod trials in PPMS and with siponimod and natalizumab in SPMS [33, 34].

Almost all ongoing clinical trials evaluating Bruton tyrosine kinase (BTK) inhibitors (remibrutinib, tolebrutinib, fenebrutinib, SAR443820) considered NfL as outcome biomarkers, suggesting a future clinical utility in assessing the effectiveness of these drugs.

Serum NfL were also employed in one trial to assess the efficacy of aHSCT compared to BMT, since two trials have documented that CSF and serum NfL were significantly decreased and remained low in MS patients that responded to aHSCT [35, 36].

In everyday clinical practice, NfLs maintain a marginal role. In orientating therapeutic decision-making, clinicians mainly rely on clinical symptoms and MRI measures. However, the use of NfL in ongoing and past clinical trials underlines the possibility of employing NfL in monitoring and evaluating the disease-modifying effect of the treatment on a single patient. Furthermore, one recent investigation conducted on 203 patients reported the usefulness of NfL in progressive MS, whereas NfLs often resulted in the only non-clinical indicator of ongoing disease activity [37]. Nevertheless, NfLs pose some significant confounding problems. Plasma NfL is negatively correlated with body mass index (BMI) and blood volume [38]. Serum NfLs are positively correlated with age, due to age-related neurodegeneration, [39], creating a significant confounding factor since progressive MS patients tend to be older patients. Moreover, a large overlap of NfL levels has been described in MS early stages of MS and controls affected by migraine and conversion disorders [8]. Finally, NfL are non-specific biomarkers of MS since their levels are elevated also in other neurological disorders, particularly neurodegenerative and infective disorders [40]. Therefore, in order for NfL measurement to be part of everyday clinical practice, validation studies, age, and concomitant disease-related normal ranges are needed; standardization of laboratory methodologies is also mandatory.

Among other biomarkers, GFAP was the most frequently adopted in ongoing clinical trials. Since CSF levels of GFAP have been associated with shorter relapse intervals and greater disabilities, GFAP levels have been adopted predominantly as outcome measure in RRMS patients (Table 3). However, CSF GFAP may help to differentiate different disease subtypes, particularly PPMS and RRMS, in their early stages, and it might represent a marker of disease severity and progression also in progressive phenotypes. Nonetheless, more preclinical studies with larger cohorts are needed to validate these findings. Furthermore, in 3 out of 4 protocols, GFAP levels were assessed in serum; it is important to consider that almost all findings about GFAP levels were obtained in the CSF of MS patients, and such findings still need to be adequately replicated in serum.

Regarding inflammatory biomarkers, their employment is even farther from validation. Few studies support the adoption of Chi3L1 and CXCL13, and they have shown contrasting results. In ongoing clinical trials, Chi3L1 was adopted as an outcome measure in RRMS patients (*n*=2) and progressive patients (SPMS *n*=1, RRMS *n*= 1), as shown in Table 3. A recent metanalyses [41] evidenced CIS patients to have higher levels of CSF Chi3L1 compared to healthy controls, suggesting an overexpression from the early phase of the disease and highlighting its potential as a prognostic biomarker. Furthermore, no significant difference in Chi3L1 levels was observed between progressive MS and RRMS patients, neither during relapse phases, suggesting that it could serve as an outcome biomarker for all MS phenotypes. In RRMS patients, natalizumab, fingolimod, mitoxantrone, and interferon beta were found to reduce CSF levels of Chi3L1 [42, 43], while glatiramer acetate and dimethylfumarate did not influence Chi3L1 levels [15, 44].

On the other hand, CXCL13 has been adopted only in one protocol as an outcome biomarker in PPMS (Table 3). In progressive phenotype, CXCL13 showed a correlation with disease activity and IgG-index and intrathecal B-cell response [21]; this may be important considering that the presence of leptomeninges infiltrating B-cells represents a culprit of neuropathology of progressive forms of MS [45]. It is also important to note that the tight correlation between CXCL13 and B-cells activity makes CXCL13 a perfect candidate to measure the therapeutic efficacy of B-cells depleting therapies. However, no specific data is available in the literature regarding ocrelizumab and ofatumumab effects on CXCL13 levels.

Both biomarkers, Chi3L1 and CXCL13, have been mostly evaluated in CSF and rarely in serum All selected protocols evaluated their levels in blood, but more validation studies to replicate what was found in CSF are still needed.

Finally, insufficient data in the literature support the employment of cytokines, such as IL-6 and IL-10, in MS clinical trials. Despite that, IL-6 was adopted in 3 clinical trials as outcome biomarker in, respectively, RRMS, SPMS, and PPMS patients (Table 3). Even if in vitro data suggest that the overexpression of proinflammatory cytokines, such as IL-6, can induce neurodegeneration [46], these evidences need to be confirmed in vivo on MS patients’ cohorts.

For a biomarker to be deemed effective for MS, as per Paul A et al. [47], it should be easily measurable, possess high sensitivity and specificity, correspond to a specific disease aspect, and be cost-efficient. Another unrealized potential is using biomarkers to pinpoint optimal treatments for individual patients [27]. New fluid biomarkers, post-validation, could be pivotal in upcoming clinical trials. Tau protein CSF levels, which are responsible for stabilizing axonal microtubules and released after neuronal damage [48], seem to directly correlate with the severity of clinical symptoms [49], quicker disease progression [50], EDSS score, and T2 lesion load in both CIS and RRMS patients [51]. It also seems that higher Tau CSF levels predict a higher risk of conversion from CIS to clinically definite MS. However, one study did not show significant differences in Tau protein CSF concentration between MS patients of all clinical subtypes and healthy controls [52]. In one study, plasma soluble CD40L (sCD40L) resulted significantly increased in SPMS patients compared to non-progressive benign MS, and the combination of plasma sCD40L with monocyte chemoattractant protein 1 (MCP1) showed great accuracy in differentiating RRMS and SPMS patients [53]. Finally, Kappa Free Light Chain (KFLC) are produced during the synthesis of antibodies by plasma cells [54] and have been found to be increased in CSF and serum of MS patients; furthermore, KFLC levels correlated with disability [55], disease [56, 57] progressions.

Fluid biomarkers, with the exception of oligoclonal bands and the light chain index, present substantial challenges in their application to MS, hindering their incorporation into future diagnostic criteria. Several key obstacles might be particularly relevant for MS. The generalizability of findings with candidate biomarker is constrained by considerable interstudy variability in preanalytical processes, including specimen collection methodologies, timing of processing, and storage conditions. Existing literature suggests that these preanalytical factors may contribute to nearly 68% of all laboratory discrepancies [58]. Moreover, the robustness of numerous studies is undermined by suboptimal design manifested in heterogeneous patient and control groups, a dearth of prospective analyses, and inadequate sample size [59]. Other factors might negatively affect the translation from bench to bedside of validated fluid biomarkers. Primarily the fact that the majority is not specific for MS since their levels in biological samples might be influenced by numerous neurological conditions. In addition, the clinical relevance of several candidate biomarkers might go beyond their validation due to a difficult application in everyday clinical practice (for example, characterization of lymphocyte intracellular vesicles and susceptibility genes to diagnostic purposes). A critical consideration is also the lack of sufficient clinical endpoints to affirm the surrogate role of these biomarkers [27]. Relapse rate might not adequately describe disease activity since it does not evidence subclinical relapses; EDSS only captures a significant progression of the disease that often occurs in a matter of years and is not able to discriminate true disease progression from relapse-dependent accumulation of disability [60]. EDSS primarily focuses on deambulation, overlooking other essential aspects such as cognitive decline when mobility becomes compromised. Considering that the majority of studies, whether prospective or retrospective, assess follow-up periods of 1–3 years, the utilization of such low-sensitivity clinical measure may pose significant limitation in the context of a chronically progressive disease like MS.

## Conclusions and limitations

Fluid biomarkers possess the potential to capture various aspects of the disease. At now, only oligoclonal bands and light chain index are adopted in clinical practice for their relevance in diagnosing MS; no fluid biomarker is validated for an early identification of different disease subtypes nor as a reliable progression biomarker. For a diagnostic biomarker to be useful, it should closely correlate with the underlying pathophysiological processes of MS. However, given our limited understanding of mechanisms underlying MS progression, pinpointing fluid biomarkers that can precisely identify MS phenotypes remains a challenge. Conversely, fluid biomarkers associated with neurodegenerative and repair mechanisms appear to be particularly promising, since they might become targets for innovative drugs tackling disease progression or be used in clinical trials to ascertain drugs neuroprotective efficacy. However, achieving this requires a focused effort to develop sensitive clinical outcomes. Furthermore, while some biomarkers have undergone retrospective validation, they still demand prospective validation in clinical trials. Specifically, the integration of these validated fluid biomarkers into a substantial number of phase 2 and 3 clinical trials is crucial.

Neurofilament light chains (NfLs) appear as the foremost biomarkers poised for integration into clinical practice. Nonetheless, additional research is needed to establish age and disease-related baseline values. As for other emerging fluid biomarkers, extensive validation studies on larger groups are essential. Additionally, establishing robust statistical correlations between measurements in cerebrospinal fluid (CSF) and peripheral blood samples is vital for their integration as outcome measures in clinical trials.

This review is characterized by several inherent limitations that warrant mention. The foremost limitation is the constrained number of clinical trials that met the eligibility criteria. With only 32 trials included, this restricted sample might not represent the entire research spectrum in the domain, thereby potentially affecting the generalizability of our conclusion. The included trials encompassed a heterogeneous population, covering varied age groups, disease phenotypes, baseline EDSS score/disability, and pharmaceutical histories. Such variability, while providing a comprehensive range of data, also introduces potential confounders that could influence interpretations concerning fluid biomarkers. A further consideration emerges from the heterogenous distribution of trial phases. While most studies were in phases 3 and 4, characterized by large cohorts and real-world relevance, four were in initial phases 1 and 2. These early-phase studies, employing fluid biomarkers, have an explorative nature, possibly limiting their congruence with advanced-phase trials.

Diagnostic criteria variation further adds to the list of limitation. Most studies employed the 2017 Revised McDonald Criteria for diagnosis, yet other used the 2010 McDonald Criteria or the 2014 Lublin Criteria. This variation introduces potential inconsistencies in patient recruitment and diagnostic rigor, which could, in turn, affect the outcomes tied to fluid biomarkers. A disparity was also noted in how fluid biomarkers were utilized as primary or secondary outcomes across the trials, presenting challenges when drawing definitive conclusions or comparing results between studies. Additionally, the review includes studies with varied pharmacological interventions, from approved second-line therapies to emerging drugs. The differential effects of these drugs on fluid biomarkers could introduce further confounding variables. Lastly, although fluid biomarkers utilization remained uniform across the trials, the designs showed significant variations, especially when considering their phases. This inconsistency, despite a shared biomarker objective, might yield varied results, analytical intricacies, and potential biases.

In light of these limitations, it is imperative to interpret the findings of this review with caution, especially when generalizing them to broader contexts or making direct comparisons across the different trial phases and diverse patient populations.
